# Effects of the Implementation of an Emergency Surgical Pattern in Patients with Rhegmatogenous Retinal Detachment: A Retrospective Observational Study

**DOI:** 10.1155/2022/4240225

**Published:** 2022-10-14

**Authors:** Ziye Chen, Kai Gao, Kunbei Lai, Wenbin Zheng, Jizhu Li, Yaping Liu, Baoyi Liu, Xiaoyue Wei, Yuan Ma, Zitong Chen, Rebiya Tuxun, Tao Li

**Affiliations:** State Key Laboratory of Ophthalmology, Zhongshan Ophthalmic Center, Sun Yat-sen University, Guangdong Provincial Key Laboratory of Ophthalmology and Visual Science, Guangdong Provincial Clinical Research Center for Ocular Diseases, Guangzhou 510060, China

## Abstract

**Background:**

To analyze the effects of the implementation of emergency surgical patterns in patients with rhegmatogenous retinal detachment (RRD) and provide evidence for promoting emergency surgical patterns for RRD.

**Methods:**

We reviewed the electronic medical records of 346 patients (348 eyes) who underwent surgical repair of RRD at the Zhongshan Ophthalmic Center in Southern China. A total of 140 patients (140 eyes) in the routine inpatient surgery group were collected at the fundus disease department between January 2019 and December 2019, and 206 patients (208 eyes) in the emergency surgery group were collected at the ophthalmic emergency department between January 2021 and December 2021. Demographics, best-corrected visual acuity (BCVA) expressed as the logarithm of the minimum angle of resolution (logMAR), the status of the macula before surgery, time to presentation, treatment interval, and postoperative BCVA measured at least three months follow-up were compared.

**Results:**

The preoperative BCVA (logMAR) of the emergency surgery group and the inpatient surgery group were 1.0 (0.4–1.7) and 1.4 (0.7–1.7), respectively, with significant differences between groups (*P* < 0.001). However, patients had a shorter time to presentation (7 days vs. 21 days, *P* < 0.001), shorter treatment interval (2 days vs. 12 days, *P* < 0.01), and significantly better postoperative BCVA (logMAR 0.5 vs. logMAR 1.0, *P* < 0.001) in the emergency surgery group than in the inpatient surgery group. There was no significant difference in primary anatomical success between the two groups (*P*=0.802). The median follow-up for the emergency surgery group and the inpatient surgery group were 6.08 months and 6.2 months, respectively, with no significant differences (*P* > 0.05).

**Conclusions:**

Patients who underwent emergency surgical patterns of RRD had better visual outcomes after surgery than patients with routine inpatient surgery, which might be attributed to a shorter duration, shorter treatment interval, and the preoperative status of the macula in the emergency surgery pattern. Emergency surgical patterns for RRD should be considered to achieve better surgical outcomes in suitable patients.

## 1. Introduction

Rhegmatogenous retinal detachment (RRD) is the most common retinal emergency that threatens vision without surgery. Research has shown that photoreceptor cell death is immediately induced as early as 12 hours and peaks at around 2–3 days after RRD, lead to irreversible vision decline [1]. While many preoperative and intraoperative prognostic factors have been studied, the strongest and most consistent predictors of visual outcomes were preoperative visual acuity and the status of the macula [2, 3]. Prompt surgery in eyes with macula-on RRD can prevent foveal detachment [4]. As for eyes with macula-off, reattaching the retina as soon as possible is the key to saving the greatest amount of visual function. Therefore, most developed countries have defined RRD as an ophthalmic emergency and implemented emergency surgery. However, due to the large population base and limited health care resources in China, RRD has not been included in ophthalmic emergencies at present. Most patients with RRD need to wait a long time before receiving routine inpatient surgery, which restricts the efficacy of treatment. Myopia is an important risk factor for RRD, and the incidence of myopia among Chinese young adults is among the highest in the world [5, 6]. Therefore, in the future, we will likely face the dilemma of a sharp increase in the number of RRDs in China [7].

In response to the above-mentioned problems and needs, Zhongshan Ophthalmic Center has built the first ophthalmic emergency department in China and has incorporated RRD into ophthalmic emergencies. In this study, we retrospectively compared the effects of the implementation of emergency surgical patterns and the conventional inpatient surgical patterns on RRD in order to provide clinical evidence to promote the emergency surgical pattern for RRD both in China and in other countries.

## 2. Methods

### 2.1. Study Design and Participants

This retrospective comparative study was conducted at the Zhongshan Ophthalmic Center (ZOC), Sun Yat-sen University, Guangzhou, China. The routine in-patient surgery group consisted of patients who underwent inpatient surgery for RRD between January 2019 and December 2019 at the ocular fundus department at ZOC. However, the coronavirus disease of 2019 has spread rapidly and caused a global pandemic since early in 2020. In order to address patients' healthcare needs during this pandemic, the ophthalmic emergency department formulated the emergency treatment protocol for RRD (supplemental file 1). The emergency surgery group consisted of patients who underwent emergency surgery for RRD between January 2021 and December 2021 in the ophthalmic emergency department at ZOC. All surgeries were performed by the same experienced vitreoretinal surgeon (T.L.).

The surgical management of RRD in this study is scleral buckling (SB) and pars plana vitrectomy (PPV). Patients with young ages and/or anteriorly located small holes underwent scleral buckling/encircling. PPV was performed in patients with an older age, pseudophakia, posterior retinal breaks, giant retinal tears, or the absence of apparent retinal breaks. The final surgical procedure was determined by the experienced retinal specialist (T.L.) considering the above multiple factors. Scleral buckling consists of subretinal fluid drainage and cryopexy with a silicone band sutured against the sclera. PPV consists of a standard three-port 23-gauge or 25-gauge PPV, fluid-air exchange, and endolaser photocoagulation with either perfluoropropane (C3F8) gas or silicone oil tamponade.

In this study, inclusion and exclusion criteria were as follows. Inclusion criteria included: (1) diagnosis of rhegmatogenous retinal detachment by slit lamp, fundus color photography, or ultrasound B-scan; (2) retinal repair surgery performed by the same surgeon; and (3) patients who were followed up for at least three months. Exclusion criteria included: (1) a history of ocular trauma and uveitis; (2) a combination of other ocular diseases, such as macular degeneration, epiretinal membrane, retinal vascular disease and choroidal detachment; and (3) previous intraocular treatment other than uneventful cataract surgery, such as intravitreal injection and retinal photocoagulation. According to these inclusion and exclusion criteria, patients with RRD treated between January 2019 and December 2019 were designated as the routine inpatient surgery group, whereas patients with RRD treated between January 2021 and December 2021 were designated as the emergency surgery group.

### 2.2. Ethics Approval

This study was approved by the Ethical Review Committee of Zhongshan Ophthalmic Center (ID: 2022KYPJ054) and adhered to the tenets of the Declaration of Helsinki for research involving human subjects. Informed consent was waived per the Institutional Review Board protocol due to the retrospective nature of this study.

### 2.3. Clinical Assessments & Data Analysis

The following data were collected for analysis: age, gender, affected eye, best-corrected visual acuity (BCVA) measurement given as the logarithm of the minimum angle of resolution (logMAR) and status of the macula, time to presentation, treatment interval, and postoperative BCVA measured at least three months after surgery, surgical methods, retinal reattachment rate after primary surgery, the number of cases requiring reoperation, and postoperative complications. The status of the macula was verified by either spectral domain optical coherence tomography (SD-OCT) before surgery or a slit lamp examination with a 90-diopter lens during the surgery.

### 2.4. Statistical Analysis

Statistical analysis was performed using IBM SPSS 20.0 software (SPSS Inc., Chicago, IL, USA). For the purposes of analysis, qualitative variables were categorized, whereas quantitative data were presented as a mean ± standard deviation (SD) or a median (interquartile range, IQR), and categorical variables were presented as frequency (No. (%)). The differences between groups were compared using a two-sample *t*-test for normally distributed continuous measures, a Mann–Whitney *U* test for nonnormally distributed continuous measures, and a chi-squared test for categorical variables. *P* < 0.05 was considered statistically significant.

The quality of this study was assessed and reported using the Strengthening the Reporting of Observational studies in Epidemiology (STROBE) checklist for an observational cohort study (supplemental file 2).

## 3. Results

### 3.1. Patient Demographics

**Figure 1:**
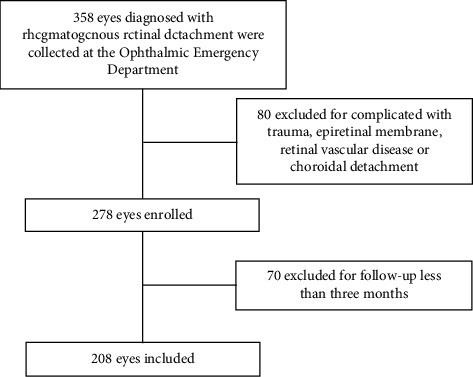
Flow chart showing the process for determining eligibility for inclusion in the emergency surgery group between January 2021 and December 2021.

**Figure 2:**
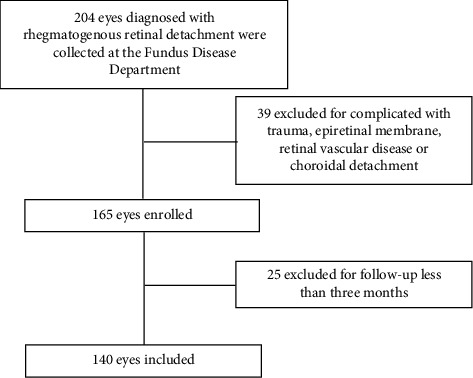
Flow chart showing the process for determining eligibility for inclusion in the inpatient surgery group between January 2019 and December 2019.

A total of 206 patients (208 eyes) were enrolled in the emergency surgery group, and 140 patients (140 eyes) were enrolled in the routine inpatient surgery group (Figures 1 and 2, respectively), according to the inclusion and exclusion criteria. Demographic data for the emergency surgery group and the inpatient surgery group are presented in Table 1. Patients had a younger age in the emergency surgery group than in the routine inpatient surgery group. According to the preoperative status of the macula, RRD was divided into two types: macula-on and macula-off. Notably, there were fewer macula-offs (65.87% vs. 77.14%, *P* < 0.05) in the emergency surgery group. All subjects were followed up at least three months after surgery. The median follow-up for the emergency surgery group and the inpatient surgery group were 6.08 months and 6.2 months, respectively, with no significant differences (*P* > 0.05).

### 3.2. Time to Presentation and Treatment Interval

**Figure 3:**
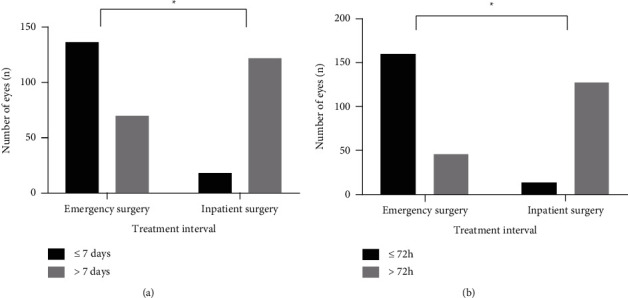
Differences in time to presentation and treatment interval in both groups. (a) Comparison of the time to the presentation of RRD between the two groups. (b) Comparison of the treatment interval between the two groups. ( ^*∗*^indicates *P* < 0.01).

The time to presentation of the patients with RRD was defined as the time from the onset of symptoms to their presentation to the ophthalmologist. The median (IQR) of time to presentation was 7 (3–12) days, and 65.87% of patients had vision loss less than or equal to 7 days in the emergency surgery group. While in the inpatient surgery group, the median (IQR) of time to presentation was 21 (14–30) days, and 12.77% of patients had vision loss less than or equal to 7 days. Compared with the inpatient surgery group, the emergency surgery group had a shorter time to presentation (*P* < 0.001).  Figure 3(a) presents the differences in time to presentation between the two groups.

The treatment interval was defined as the patient's waiting time between the time of diagnosis and surgery. In the emergency surgery group, the median (IQR) of the treatment interval was 2 (1–3) days, and the number of eyes with a treatment interval of ≤72 h was 161 (77.40%). While for the inpatient surgery group, the median (IQR) of the treatment interval was 12 (8–19) days, and there were only 14 eyes (10.0%) with a treatment interval of ≤72 h. Compared with the inpatient surgery group, the emergency surgery group had a significantly shorter treatment interval (*P* < 0.01), as shown in Figure 3(b).

### 3.3. Visual Outcomes

In the emergency surgery group, 55 of 208 (26.44%) eyes underwent SB, and the other 151 eyes were repaired with either PPV (151 of 208, 72.60%) or combined PPV/SB (2 of 208, 0.96%). In the Inpatient surgery group, 20 of 140 (14.29%) eyes underwent SB, and the other 117 eyes were repaired with either PPV (117 of 140, 83.57%) or combined PPV/SB (3 of 140, 2.14%).

**Figure 4:**
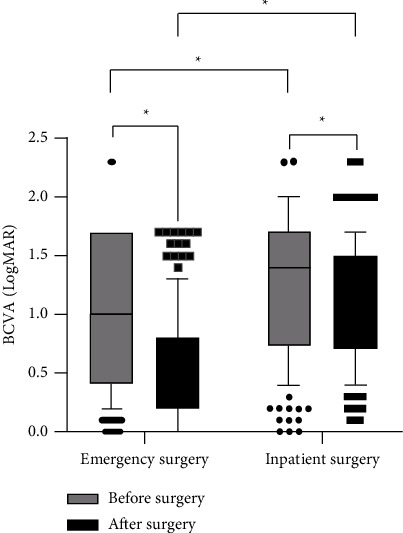
Changes of BCVA after RRD surgery in the emergency surgery group and the inpatient surgery group ( ^*∗*^indicates *P* < 0.01). RRD = rhegmatogenous retinal detachment. BCVA = best corrected visual acuity.

Overall visual outcomes are detailed in Figure 4. At the last follow-up, the median (IQR) of BCVA in the emergency surgery group improved from logMAR 1.0 (0.4–1.7) to logMAR 0.5 (0.2–0.8) (*P*  <  0.001), indicating significant visual improvement after emergency surgery. In the inpatient surgery group, the median (IQR) of BCVA increased from logMAR 1.4 (0.7–1.7) to logMAR 1.0 (0.7–1.5) with statistically significant differences (*P* < 0.001). Moreover, the postoperative BCVA of the emergency surgery group was significantly better than that of the inpatient surgery group (*P* < 0.001).

**Figure 5:**
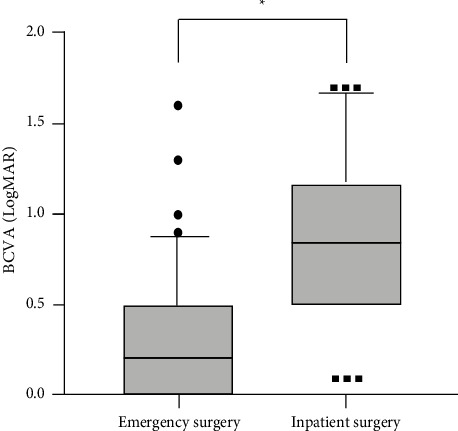
Differences in postoperative BCVA between the emergency surgery group and the inpatient surgery group among RRD eyes with macula-on ( ^*∗*^indicates *P* < 0.001) RRD = rhegmatogenous retinal detachment. BCVA = best corrected visual acuity.

**Figure 6:**
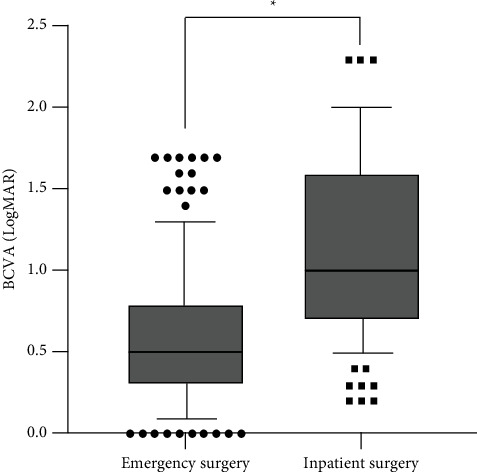
Differences in postoperative BCVA between the emergency surgery group and the inpatient surgery group among RRD eyes with macula-off ( ^*∗*^indicates *P* < 0.001). RRD = rhegmatogenous retinal detachment. BCVA = best corrected visual acuity.

Among all the RRD eyes with macula-on, the cases in the emergency surgery group had significantly better postoperative BCVA (median BCVA, logMAR 0.2 (0–0.5)) than those in the inpatient surgery group (median BCVA, logMAR 0.8 (0.5–1.2); *P*  <  0.001), as shown in Figure 5. Likewise, among all the RRD eyes with macula-off, the cases in the emergency surgery group also had significantly better postoperative BCVA (median BCVA, logMAR 0.5 (0.3–0.8)) than those in the inpatient surgery group (median BCVA, logMAR 1.0 (0.7–1.6); *P* < 0.001), as shown in Figure 6.

During the follow-up, there were 11 eyes that had a recurrent retinal detachment in the emergency surgery group. Among them, 5 eyes received PPV with C3F8 gas tamponade, 5 eyes received scleral buckling, and the other eye received PPV with silicone oil tamponade. The reasons for the recurrence of retinal detachment were the rapid absorption of the gas (C3F8), proliferative vitreoretinopathy (PVR), and a new hole that occurred after the primary surgery. Recurrent retinal detachment occurred in 6 eyes in the inpatient surgery group. Among them, 4 eyes received scleral buckling, and the other 2 eyes received PPV with silicone oil tamponade. Recurrent retinal detachment occurred due to PVR, and a new hole occurred in the follow-up. There was no significant difference in primary anatomical success between the emergency surgery group and the inpatient surgery group (94.71% vs. 95.71%, *P*=0.802). After reoperation, retinal reattachment was achieved in all eyes in the two groups. Besides, during the follow-up, there were 32 eyes (15.4%) that had significant cataract progression in the emergency surgery group and 33 eyes (23.57%) in the inpatient group with no significant differences (*P* > 0.05). It is noteworthy that all these cases with significant cataract progression in both groups were treated with PPV with silicone oil tamponade.

## 4. Discussion

Recent improvements in surgical techniques, instruments, and surgeons' experience in RRD surgery have resulted in a final anatomic success rate of at least 90% [8]. However, we found that even after retinal reattachment, few patients could have a good postoperative visual outcome, which was closely related to the duration of retinal detachment. Some studies have proven that receiving surgery as soon as possible after the onset of RRD and reattaching the retina was crucial to achieving a better visual outcome to the greatest extent [9–11] However, there is insufficient evidence on whether emergency surgery is beneficial to the prognosis of RRD in the real clinical setting, especially in China.

With the increasing incidence of myopia in China, there will be more and more RRD patients, which poses new challenges to traditional ophthalmic diagnosis and treatment services. Various subspecialties in ophthalmology and the complex registration system in traditional clinics make it very hard for patients to distinguish the department to which they should go. Therefore, these difficulties prevent prompt presentation to the retinal specialist and eventually delay the repair surgery of RRD, which can result in worse visual outcomes. In cases of quick emergency triage services and rapid consultations with retinal surgeons, RRD patients can be treated in time. In our present study, the emergency surgery group had a significantly shorter time to presentation as well as a shorter treatment interval than the inpatient surgery group, which was more likely attributed to the better visual outcomes.

RRD may rapidly progress to involve the macula during the scheduled treatment interval. Therefore, RRD eyes with macula-on should undergo surgery as soon as possible to achieve ideal anatomic and functional outcomes. Ho et al. [9] included 82 RRD cases with macula-on in their study. Of the 82 cases, 11 cases demonstrated progression of RRD when they were waiting for surgery, of which three were found to involve the macula at the time of the operation, displaying a progression of RRD with an average rate of 1.88 disc diameters/day.

In our present study, 161 eyes (77.40%) underwent surgery within 72 h, and the median treatment interval was only 2 (1–3) days in the emergency surgery group. While in the inpatient surgery group, there were only 14 eyes (10.00%) that underwent surgery within 72 h, and the median treatment interval was 12 (8–19) days. Under the traditional inpatient surgery pattern, long queues for surgery with wait times of nearly two weeks or even one month definitely delay the treatment and affect postoperative visual function. However, patients with emergency surgery patterns could receive prompt surgery with a shorter treatment interval, which would contribute to a better visual outcome.

Previous studies have shown that for RRD with macula-off, the longer the duration, the worse the postoperative visual function. Williamson et al. [10] analyzed a series of 325 cases of RRD with macula-off and found that cases with macula-off of less than 3 days had better visual outcomes, compared with cases with macula-off of more than 3 days. In another retrospective study by Lee et al. [11], macula that was detached for more than 8 days was significantly associated with poorer postoperative visual outcomes, and there was no significant difference in postoperative visual acuity between macula-on cases and macula-off cases with less than three days. We found that the emergency surgery patterns could enable RRD patients to undergo surgery in a timely manner, thus achieving better visual outcomes than the inpatient surgical pattern. In our present study, the median treatment interval in the emergency surgery group is significantly shorter than that in the routine inpatient surgery group (2 [1–3] vs. 12 [8–19] days). Therefore, it is worth mentioning that the optimal treatment window for both macula-on and macula-off RRD patients needs to be narrowed down in further prospective studies in the future.

Our findings have important clinical implications for the implementation of an emergency surgical pattern of RRD in China. However, our study had several limitations. First, we did not precisely compare and analyze the location of retinal detachment, the number of retinal tears, and the grades of PVR in all cases. Second, the duration of follow-up in this study was relatively limited. Longer follow-ups are needed to evaluate the long-term prognosis of RRD in both groups. Third, as the OCT could evaluate the structural integrity of the retina before and after surgery, the likely explanation for why some patients have little improvement in visual acuity postoperatively requires further studies with the help of the OCT examination.

## 5. Conclusions

The results of this study confirmed that patients with RRD who underwent emergency surgery achieved significantly improved visual outcomes. Therefore, surgical intervention for RRD in an emergency setting is worthy and valuable enough to be promoted in order to get better postoperative visual function.

## Figures and Tables

**Table 1:** Clinical and demographics of the emergency surgery group and the inpatient surgery group.

Characteristics	Emergency surgery group	Inpatient surgery group	*P* value
	Mean ± standard deviation or no. (%)	Mean ± standard deviation or no. (%)	
Patients/no. of eyes	206/208	140/140	

Age, years	45.33 ± 14.68	50.77 ± 14.11	<0.001^*∗*^

Gender			
Male	106 (51.46)	91 (65.00)	0.01^*∗*^
Female	100 (48.54)	49 (35.00)	

Affected eye			
Right eye	119 (57.21)	80 (57.14)	0.99
Left eye	89 (42.79)	60 (42.86)	

Macula status			
Macula-on	71 (34.13)	32 (22.86)	0.03^*∗*^
Macula-off	137 (65.87)	108 (77.14)	

^*∗*^Indicates statistical significance (*P* < 0.05).

## Data Availability

The datasets obtained and/or analyzed during the current study are available from the corresponding author upon reasonable request.

## Supplementary Materials

Supplementary Materials Supplemental file 1. The diagnosis and treatment protocol of patients with RRD in the Ophthalmic Emergency Department in Zhongshan Ophthalmic Center. RRD = rhegmatogenous retinal detachment. Supplemental file 2. Completed STROBE cohort checklist.
