# Supplementary material for: Effects of the Implementation of an Emergency Surgical Pattern in Patients with Rhegmatogenous Retinal Detachment: A Retrospective Observational Study
Source: J Ophthalmol. 2022 Oct 14;2022:4240225. doi: 10.1155/2022/4240225 (PMC9586816; doi:10.1155/2022/4240225)
Supplement: Supplementary Materials — Supplemental file 1. The diagnosis and treatment protocol of patients with RRD in the Ophthalmic Emergency Department in Zhongshan Ophthalmic Center. RRD = rhegmatogenous retinal detachment. Supplemental file 2. Completed STROBE cohort checklist. [file 4240225.f1.zip › Supplemental file 1.pdf]

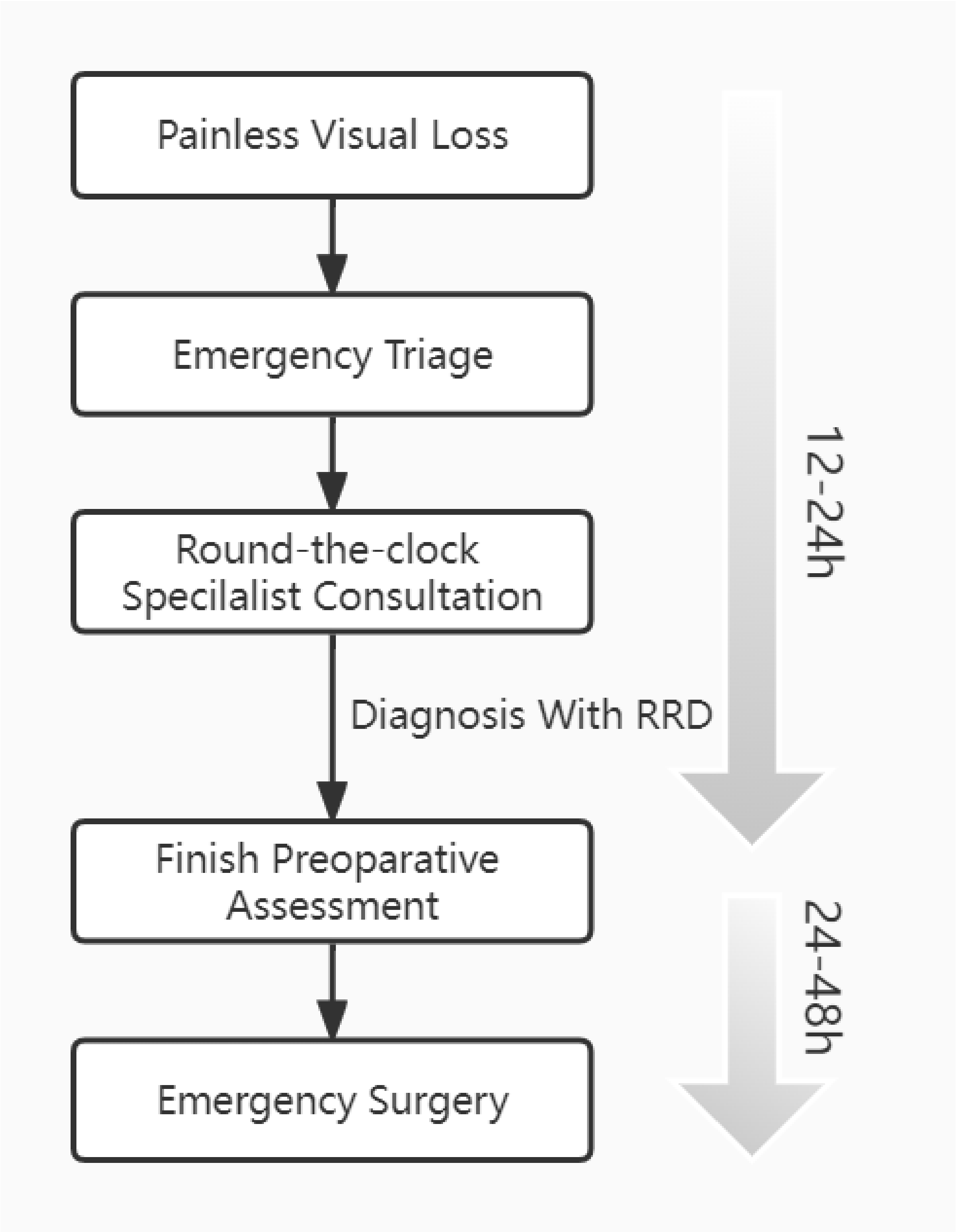
Painless Visual Loss
Emergency Triage
Round-the-clock Specilalist Consultation
Diagnosis With RRD
Finish Preoparative Assessment
Emergency Surgery
12-24h
24-48h
